# Agomelatine Prevents Amyloid Plaque Deposition, Tau Phosphorylation, and Neuroinflammation in APP/PS1 Mice

**DOI:** 10.3389/fnagi.2021.766410

**Published:** 2022-01-27

**Authors:** Xiao-bo Yang, Heng-bing Zu, Yong-fei Zhao, Kai Yao

**Affiliations:** Department of Neurology, Jinshan Hospital Affiliated to Fudan University, Shanghai, China

**Keywords:** agomelatine, Alzheimer’s disease, Aβ, tau hyperphosphorylation, neuroinflammation

## Abstract

Agomelatine, an agonist of melatonergic MT1 and MT2 receptors and a selective 5-hydroxytryptamine 2C receptor antagonist, is widely applied in treating depression and insomnia symptoms in several neurogenerative diseases. However, the neuroprotective effect of agomelatine in Alzheimer’s disease (AD) is less known. In this study, a total of 30 mice were randomly divided into three groups, namely, wild type (WT), APP/PS1, and agomelatine (50 mg/kg). After 30 days, the Morris water maze was performed to test the cognitive ability of mice. Then, all mice were sacrificed, and the hippocampus tissues were collected for ELISA, Western blot, and immunofluorescence analysis. In this study, we found that agomelatine attenuated spatial memory deficit, amyloid-β (Aβ) deposition, tau phosphorylation, and neuroinflammation in the hippocampus of APP/PS1 mice. Further study demonstrated that agomelatine treatment upregulated the protein expression of DHCR24 and downregulated P-Akt, P-mTOR, p-p70s6k, Hes1, and Notch1 expression. In summary, our results identified that agomelatine could improve cognitive impairment and ameliorate AD-like pathology in APP/PS1 mice *via* activating DHCR24 signaling and inhibiting Akt/mTOR and Hes1/Notch1 signaling pathway. Agomelatine may become a promising drug candidate in the therapy of AD.

## Introduction

As the most common progressive neurogenerative disease, the etiology of Alzheimer’s disease (AD) is not clear. Generally, the extracellular deposition of amyloid-beta (Aβ) plaques and the accumulation of intracellular neurofibrillary tangles (NFTs) are the core pathophysiology of AD (Scheltens et al., 2016). In the pathological state, tau protein hyperphosphorylation could induce tau aggregation and the formation of NFT, aggravate synaptic impairment, and promote neurodegeneration (Ahmed et al., 2014; Gao et al., 2018). In contrast, in recent years, more and more studies demonstrated that neuroinflammation played a vital role in the onset and development of AD. At the early stage of AD, neuroinflammation, characterized by the activation of microglia and astrocyte, exerts beneficial effects, including Aβ cleavage (Hansen et al., 2018) and tissue repair (Mecha et al., 2015). With the progression of AD, aberrant and excessive inflammatory response, accompanied by the change of microglial polarization, provides a toxic effect, resulting in neuronal injury, Aβ deposition (Krabbe et al., 2013), tau hyperphosphorylation (Bhaskar et al., 2010), and synaptic loss (Hansen et al., 2018). Modulating cerebral neuroinflammation and promoting microglial polarization from pro-inflammatory M1 to anti-inflammatory M2 phenotype could suppress the pathological damage of AD *in vitro* and *in vivo* (Yao and Zu, 2020). In conclusion, exploring novel and practical therapeutic approaches targeting Aβ, NFTs, and neuroinflammation may become a good orientation in the AD field.

There is a close link between AD and depression. As the most prevalent comorbidity of AD, depression shares some common etiologies with AD like nitrosative stress (Nicholas, 2007). Antidepressant agomelatine, an agonist of MT1 and MT2 melatonin receptors and 5-hydroxytryptamine 2C receptor antagonist, is widely used for treating depression and insomnia in several neurogenerative diseases, including Parkinson’s disease (Green, 2011; Norman and Olver, 2019). However, the effect of agomelatine in AD treatment is less known. The previous report showed that agomelatine injection could improve behavioral deficits and alleviate hippocampal Aβ deposition in streptozotocin (STZ)-induced AD rat model (Ilieva et al., 2019). Our previous study also found that after agomelatine intervention, tau protein hyperphosphorylation and oxidative injury obviously decreased in Aβ_25–35_-treated BV-2 cells (Yao et al., 2019). Besides, some recent studies suggested that agomelatine may exert anti-inflammatory properties *in vitro* and *in vivo* (Cheng et al., 2020; Savran et al., 2020). Although less evidence has focused on the effect of agomelatine in AD therapy, there are also limitations. First, the effect of agomelatine on AD-like pathologies, such as tau protein hyperphosphorylation and neuroinflammation, is still not clear. Second, the downstream signaling mechanisms are not fully understood.

In this study, we identified that antidepressant agomelatine improved cognitive deficits and ameliorated AD-like pathologies, including Aβ deposition, tau protein hyperphosphorylation, and neuroinflammation in APP/PS1 mice, *via* activating DHCR24 signaling and inhibiting Akt/mTOR and Hes1/Notch1 signaling pathway. The results indicated that agomelatine could be invoked as a potential therapeutic strategy for AD disease.

## Materials and Methods

### Materials

Agomelatine (#A124691) was obtained from Aladdin Company (China); the primary antibodies against phospho-Akt (Ser473) (#4060S), phospho-mTOR (#5536S), mTOR (#2983S), phospho-p70 S6 kinase (#9234), p70 S6 kinase (#2708), and Hes1 (#11988S) were purchased from Cell Signaling Technology (United States). The primary antibodies against amyloid-β (#60342-1-Ig), DHCR24 (#10471-1-AP), Akt (10176-2-AP), Arginase-1 (#16001-1-AP), GFAP (#60190-1-Ig), Tau (#10274-1-AP), and Notch1 (#20687-1-AP) were purchased from Proteintech Group (United States). The primary antibodies against phospho-tau (Thr181) (#ab75679), phospho-tau (S396) (#ab109390), and i-NOS (#ab178945) were purchased from Abcam Company (United States). Mouse Interleukin 1 Beta (IL-1β) ELISA Kit (E-EL-M0037c), Transforming Growth Factor Beta 1 (TGF-β1) ELISA Kit (E-EL-0162c), Mouse Interleukin 4 (IL-4) ELISA Kit (E-EL-M0043c), Mouse Tumor Necrosis Factor Alpha (TNF-α) ELISA Kit (E-EL-M0049c), Mouse Amyloid Beta 1-40 (Aβ_1–40_) ELISA Kit (E-EL-M3009), and Mouse Amyloid Beta 1-42 (Aβ1-42) ELISA Kit (E-EL-M3010) were obtained from Elabscience Company (China).

### Animal Experiments

Female APP/PS1 transgenic mice (6-months old; body weight 18–22 g) were purchased from Changzhou Cavens Lab Animal Co. Ltd. (SPF, SCXK, 2016-0010, Jiangsu, China). The wild-type (WT) C57BL/6 mice were purchased from China Three Gorges University (SPF, SYXK, 2018-0104, Hubei, China). All animals were fed in standard conditions of 12 h of light/day at a room temperature of 20–22°C. Twenty female APP/PS1 transgenic mice were randomly divided into the APP/PS1 group and the agomelatine-treated group. A total of 10 WT mice were invoked as a control group. In the agomelatine-treated group, mice were intraperitoneally injected with agomelatine (50 mg/kg/day) for 30 days.

### Morris Water Maze

After administration, the spatial learning, and memory performance of the mice was tested by the Morris water maze (MWM) assay. The MWM was utilized to analyze the long-term memory performance of animals. Briefly, the mice were subjected to the maze per day for 5 days. The place navigation experiment was performed from day 1 to day 4. Consequently, the probe trial was carried out on day 5. In the navigation experiment, mice were placed in each quadrant in turn, faced to the wall, and allowed to freely swim until they reached the platform for a maximum period of 120 s. In the probe trial, the platform was removed, and the animals were located in quadrant IV from the farthest to the target quadrant (quadrant II). During the test, the time spent in the target quadrant, the swimming track, and the covered distance were recorded. Once the MWM test was completed, all mice were subsequently euthanized and sacrificed, and then, hippocampal tissues were immediately separated for later analysis.

### Enzyme-Linked Immunosorbent Assay

Enzyme-linked immunosorbent assay (ELISA) was utilized to analyze the content of Aβ_1–40_, Aβ_1–42_, IL-1β, TNF-α, IL-4, and TGF-β according to the instructions of the manufacturer. Briefly, the brain tissue was first lysed on ice for 20 min, then the mixture was centrifuged, and the supernatants were collected. Second, the corresponding antibody was added into the micro ELISA plate with the sample for incubation for 90 min, and subsequently, the biotinylated detection antibody working solution was incubated for 1 h at 37°C. Third, the HRP conjugate working solution was mixed for 30 min, and then, a substrate reagent was incubated for 15 min at 37°C. Finally, the stop solution was added. The plate was observed by a microplate reader at 450 nm.

### Western Blot

The hippocampal tissues of mice were dissected and homogenized in radioimmunoprecipitation assay (RIPA) buffer on ice for 15 min. Then, the tissues were centrifuged, and the supernatant of brain lysates was used for Western blot. First, total protein concentrations were analyzed using bicinchoninic acid (BCA) assay. Samples were prepared in sample buffer and heated to 95°C for 5 min. Second, equal amounts of lysates were fractionated by sodium dodecyl sulfate-polyacrylamide gels and electrotransferred onto nitrocellulose membranes. Gels were run at a constant voltage (80–120 mv) for 1.5 h for maximum separation, and then, the wet transfer was performed for 90–150 min at constant current (200–300 mA) using polyvinylidene difluoride membrane presoaked in methanol. The membrane was blocked in 5% milk in 0.2% PBST and then washed three times in PBST for 15 min each. After overnight incubation at 4°C with the primary antibodies [P-tau (S396) 1:1,000, P-tau (T181) 1:500, tau 1:1,000, i-NOS 1:1,000, arginase-1 1:1,000, DHCR24 1:800, P-Akt 1:1,000, Akt 1:1,000, p-mTOR (Ser2448) 1:1,000, mTOR 1:1,000, p-p70s6k (Thr389) 1:1,000, p70s6k 1:1,000, Hes1 1:1,000, Notch1 1:1,000, GFAP 1:2,000, GAPDH 1:5,000], the blots were washed and incubated into corresponding HRP-conjugated secondary antibodies for 1 h. Chemiluminescent (Bio-Rad, Hercules, CA, United States) detection was then used to detect the expression of each protein. GAPDH levels served as internal loading controls.

### Immunofluorescence

The immunofluorescence assay was performed as reported in a study by Yao and Zhao (2018). Briefly, brain tissues were embedded in Tissue-Tek O.C.T Compound (Sakura Finetek United States, Inc., United States), frozen in liquid nitrogen, and sectioned and stored at −20°C. Then, the brain sections were fixed with 4% PFA, thoroughly washed, and incubated with 5% BSA and 0.1% Triton X-100 at room temperature (RT). Next, the primary antibodies [Aβ 1:50, P-tau (T181) 1:50, GFAP 1:200] were added into samples for incubation overnight at 4°C, following that the fluorescent secondary antibodies (CY3 Conjugated AffiniPure Goat anti-rabbit IgG and anti-mose IgG 1:100) were incubated for 60 min at RT. Finally, DAPI was added for nuclei staining, and samples were observed under a fluorescence microscope (Olympus BX60, Center Valley, PA, United States). The percentage of the Aβ plaque area and the mean fluorescence intensity of P-tau (T181) and GFAP were analyzed using ImageJ software.

### Statistical Analysis

Statistical analysis was performed by GraphPad Prism 8.0 software. All data were expressed as means ± SEM. The Shapiro-Wilk test was applied to examine normal distribution, and the Brown-Forsythe’s test was employed to check the homogeneity of variances. Data with normal distribution were analyzed by one-way ANOVA with Bonferroni’s multiple test for equal variance or Brown-Forsythe and Welch ANOVA tests for unequal variances. Otherwise, the non-parametric Kruskal-Wallis test was applied when the normality of data was not satisfied. *P* < 0.05 and *P* < 0.01 were considered as statistically significant.

## Results

### Agomelatine Improves the Performance of Morris Water Maze in APP/PS1 Mice

**FIGURE 1:**
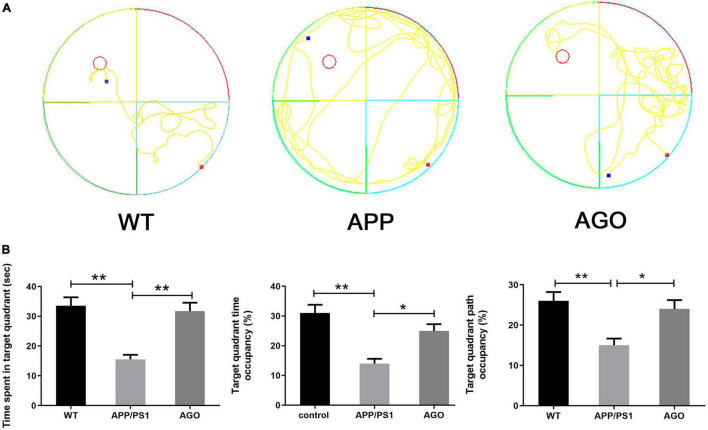
Effects of agomelatine on the spatial learning and memory performance in APP/PS1 mice. In the agomelatine-treated group, mice were intraperitoneally injected with agomelatine (50 mg/kg/day) for 30 days. **(A)** Performance of Morris water maze in APP/PS1 mice. **(B)** Measurement of the time spent in target quadrant, target quadrant time occupancy, and path length occupancy in the target quadrant by Morris water maze. Values are represented as the mean ± SEM (*n* = 10). *P < 0.05; **P < 0.01.

The MWM was carried out to identify the spatial learning and memory performance of mice. First, as shown in Figures 1A,B, as compared with the WT group, the time spent in the target quadrant, the target quadrant time, and the path occupancy obviously decreased in the APP/PS1 group (APP/PS1: 15.51 ± 4.897 s, WT: 33.52 ± 9.06 s, *P* < 0.0001; APP/PS1: 14 ± 5.16%, WT: 31 ± 8.756%, *P* = 0.0003; APP/PS1: 15 ± 5.27%, WT: 26 ± 6.99%, *P* = 0.0028). The above parameters could be partly reversed by agomelatine intervention (AGO: 31.71 ± 8.97 s, *P* = 0.0003; AGO: 25 ± 7.07%, *P* = 0.0213; AGO: 24 ± 6.99%, *P* = 0.0126). However, although agomelatine could partly decrease the escape latency and increase the crosses of the platform in APP/PS1 mice, the difference didn’t reach the statistic difference. The reason may be related to the dose and duration of agomelatine and need further studies. Generally, the data above exhibited that antidepressant agomelatine had the potential to improve behavioral ability in AD-transgenic animal model.

### Agomelatine Alleviates Aβ Deposition and Tau Hyperphosphorylation in the Hippocampus of APP/PS1 Mice

**FIGURE 2:**
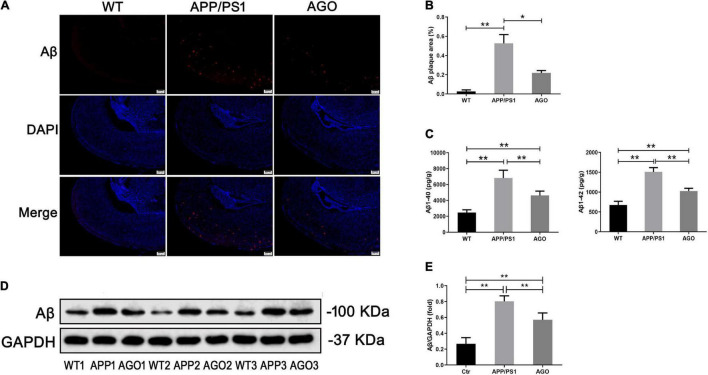
Effects of agomelatine on Aβ deposition in APP/PS1 mice. In the agomelatine-treated group, mice were intraperitoneally injected with agomelatine (50 mg/kg/day) for 30 days. **(A,B)** Measurement of Aβ expression by immunofluorescence (40×, scale bars = 200 μm) (*n* = 3). **(C)** Measurement of Aβ content by ELISA (*n* = 5). **(D,E)** Measurement of Aβ expression by Western blot (*n* = 5). Values are represented as the mean ± SEM. *P < 0.05; **P < 0.01.

Amyloid-β deposition and tau hyperphosphorylation were the essential pathological impairments of AD. First, as shown in Figures 2A,B, immunofluorescence assay of the hippocampus demonstrated a higher Aβ expression in APP/PS1 mice than in WT group (APP/PS1: 0.53 ± 0.16%, WT: 0.03 ± 0.02%, *P* = 0.002), which could be rescued by agomelatine intervention (AGO: 0.22 ± 0.04%, *P* = 0.023). Second, ELISA test also identified that the elevation of hippocampal Aβ_1–40_ and Aβ_1–42_ levels in APP/PS1 mice was abrogated upon agomelatine treatment (*P* < 0.05) (AGO: 4,621 ± 545.1 pg/g, APP/PS1: 6,810 ± 976.2 pg/g, *P* = 0.0008; AGO: 1,028 ± 67.83 pg/g, APP/PS1: 1,510 ± 106.4 pg/g, *P* < 0.0001) (Figure 2C). Then, the Western blot analysis showed that agomelatine injection could downregulate the protein expression of Aβ in the hippocampus of APP/PS1 mice (AGO: 0.57 ± 0.09, APP/PS1: 0.80 ± 0.07, *P* = 0.0014) (Figures 2D,E).

**FIGURE 3:**
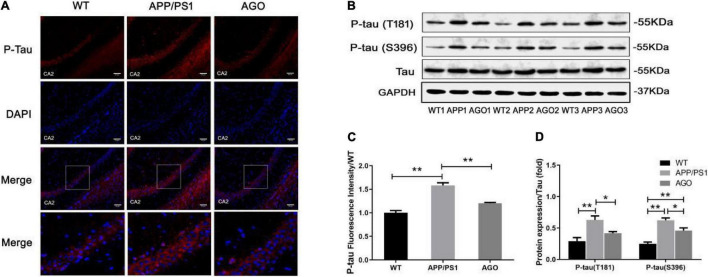
Effect of agomelatine on tau protein hyperphosphorylation in APP/PS1 mice. In the agomelatine-treated group, mice were intraperitoneally injected with agomelatine (50 mg/kg/day) for 30 days. **(A,C)** Measurement of P-tau (T181) by immunofluorescence (200×, scale bars = 50 μm) (*n* = 3). **(B,D)** Measurement of P-tau (T181) and P-tau (S396) by Western blot (*n* = 5). Values are represented as the mean ± SEM. *P < 0.05; **P < 0.01.

Moreover, the P-tau expression was analyzed using Western blot and immunofluorescence. As shown in Figures 3B,D, the protein expression of P-tau (T181) and P-tau (S396) was obviously upregulated in APP/PS1 group as compared with the WT group (APP/PS1: 0.63 ± 0.14, WT: 0.29 ± 0.13, *P* = 0.0019; APP/PS1: 0.62 ± 0.08, WT: 0.25 ± 0.06, *P* < 0.0001). The effect could be partly reversed by agomelatine treatment (AGO: 0.42 ± 0.06, *P* = 0.0407; AGO: 0.46 ± 0.09, *P* = 0.0209). Furthermore, the immunofluorescence assay also revealed that agomelatine could decrease hippocampal P-tau (T181) expression in APP/PS1 mice (AGO: 1.20 ± 0.03, APP/PS1: 1.59 ± 0.10, *P* = 0.0029) (Figures 3A,C). Thus, the results indicated that agomelatine had great potential to be a promising drug for AD treatment by diminishing Aβ deposition and tau hyperphosphorylation.

### Agomelatine Reduces the Neuroinflammatory Response in the Hippocampus of APP/PS1 Mice

**FIGURE 4:**
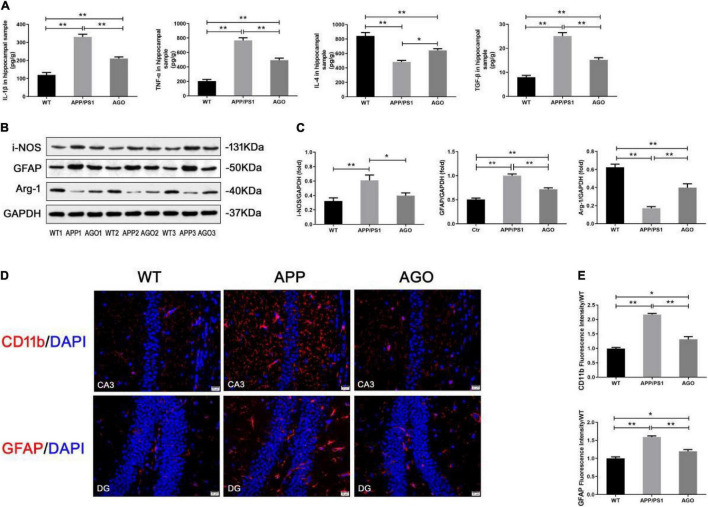
Effect of agomelatine on neuroinflammation in APP/PS1 mice. In the agomelatine-treated group, mice were intraperitoneally injected with agomelatine (50 mg/kg/day) for 30 days. **(A)** Measurement of IL-1β, TNF-α, IL-4, and TGF-β by ELISA (*n* = 5). **(B,C)** Measurement of i-NOS and arginase-1 by Western blot (*n* = 5). **(B–E)** Measurement of CD11b and GFAP by Western blot (*n* = 5) and immunofluorescence (400×, scale bars = 20 μm) (*n* = 3). Values are represented as the mean ± SEM. *P < 0.05; **P < 0.01.

Aberrant and excessive neuroinflammation was reported to accelerate the development of AD, induced by the activation of cerebral microglia and astrocyte (Yao and Zu, 2020). As shown in Figure 4A, the ELISA analysis demonstrated that the content of pro-inflammatory cytokines (IL-β and TNF-α) significantly increased (APP/PS1: 331.1 ± 32.12 pg/g, WT: 119.5 ± 31.23 pg/g, *P* < 0.0001; APP/PS1: 767.7 ± 80.05 pg/g, WT: 203.8 ± 49.87 pg/g, *P* < 0.0001) and anti-inflammatory cytokines (IL-4) decreased (APP/PS1: 483 ± 45.65 pg/g, WT: 842.8 ± 102.8, *P* < 0.0001) in the hippocampus of APP/PS1 mice when comparing with WT group. This pro-inflammatory response could be alleviated by agomelatine injection (AGO: 210.8 ± 19.46 pg/g, *P* < 0.0001; AGO: 494.6 ± 60.21 pg/g, *P* < 0.0001; AGO: 644 ± 49.41 pg/g, *P* = 0.0111). Interestingly, TGF-β, always considered as an inflammatory inhibitor, was shown to increase in APP/PS1 group (APP/PS1: 25.12 ± 3.05 pg/g, WT: 7.96 ± 1.63 pg/g, *P* < 0.0001), which was downregulated by agomelatine (AGO: 15.22 ± 1.95 pg/g, *P* < 0.0001). The related mechanism needs to be discussed further.

Then, the effect of agomelatine on hippocampal glia (microglia and astrocyte) activation was investigated. It is reported that promoting microglia polarization switch from pro-inflammatory M1 to anti-inflammatory M2 phenotype inhibited neuroinflammation and ameliorate pathological injury in AD (Yao and Zu, 2020). First, immunofluorescence test demonstrated that hippocampal CD11b expression increased in APP/PS1 group (APP/PS1: 2.17 ± 0.07, WT: 0.99 ± 0.07, *P* < 0.0001) indicated a significant microglial activation in the hippocampus of APP/PS1 mice. The pro-inflammatory response could be partly reversed by agomlatine (AGO: 1.31 ± 0.16, *P* = 0.0002) (Figures 4D,E). Second, the results of Western blot showed that, as compared with the WT group, the protein expression of M1 microglial hallmark (i-NOS) was obviously upregulated (APP/PS1: 0.61 ± 0.16, WT: 0.32 ± 0.10, *P* = 0.0085), and the M2 microglial hallmark (arginase-1) expression was downregulated in APP/PS1 mice (APP/PS1: 0.17 ± 0.04, WT: 0.62 ± 0.08, *P* < 0.0001). Agomelatine could promote microglial polarization transformation from M1 to M2 phenotype by increasing arginase-1 expression (AGO: 0.40 ± 0.09, *P* = 0.0013) and reducing i-NOS expression (AGO: 0.39 ± 0.08, *P* = 0.0497) (Figures 4B,C).

In contrast, Western blot and immunofluorescence analyses exhibited the significant expression of the GFAP, an astrocyte hallmark, in APP/PS1 group than WT group (APP/PS1: 1.00 ± 0.08, WT: 0.51 ± 0.06, *P* < 0.0001; APP/PS1: 1.59 ± 0.05, WT: 1.00 ± 0.07, *P* = 0.0002). The abovementioned parameters could be reversed by agomelatine treatment (AGO: 0.71 ± 0.07, *P* = 0.0001; AGO: 1.20 ± 0.08, *P* = 0.0014) (Figures 4B–E). Therefore, our data suggested that agomelatine could suppress hippocampal neuroinflammation in APP/PS1 mice, probably through modulating microglia polarization and astrocyte activation.

### The Neuroprotective Molecular Mechanism of Agomelatine in APP/PS1 Mice

**FIGURE 5:**
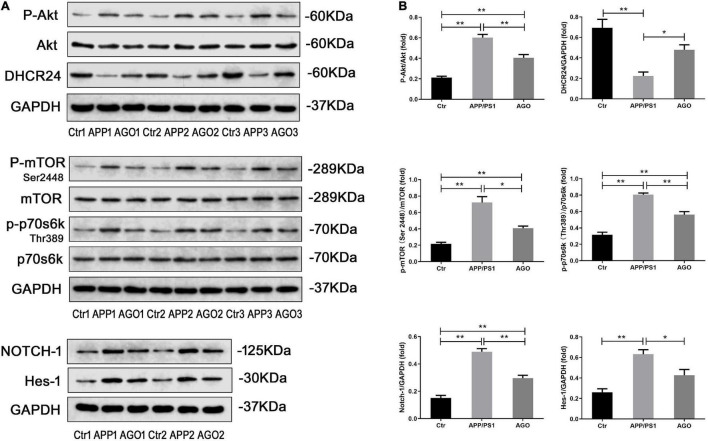
The neuroprotective mechanism of agomelatine in APP/PS1 mice. In the agomelatine-treated group, mice were intraperitoneally injected with agomelatine (50 mg/kg/day) for 30 days. **(A,B)** Measurement of P-Akt, Akt, P-GSK3β, GSK3β, p-mTOR, mTOR, p-p70s6k, p70s6k, DHCR24, Hes-1, and Notch1 expression by Western blot (*n* = 5). Values are represented as the mean ± SEM. *P < 0.05; **P < 0.01.

Previous studies demonstrated that Akt/mTOR/p70s6k signaling, Hes1/Notch1 signaling, and DHCR24 are importantly involved in the pathophysiology of AD (Crameri et al., 2006; Ho et al., 2020; Yao and Zu, 2020). In this study, we discussed the neuroprotective molecular mechanism of agomelatine in APP/PS1 mice. As shown in Figures 5A,B, the ratios of P-Akt/Akt, P-mTOR/mOTR, and p-p70s6k/p70s6k, the protein expression of Hes1 and Notch1 obviously increased in APP/PS1 group as compared with the WT group (APP/PS1: 0.60 ± 0.07, WT: 0.21 ± 0.03, *P* < 0.0001; APP/PS1: 0.72 ± 0.16, WT: 0.22 ± 0.04, *P* = 0.0038; APP/PS1:0.81 ± 0.04, WT: 0.32 ± 0.07, *P* < 0.0001; APP/PS1: 0.63 ± 0.10, WT: 0.26 ± 0.07, *P* = 0.0003; APP/PS1: 0.49 ± 0.05, WT: 0.15 ± 0.04, *P* < 0.0001); moreover, DHCR expression decreased (APP/PS1: 0.22 ± 0.09, WT: 0.69 ± 0.18, *P* = 0.0003). The effect above could be significantly reversed by agomelatine treatment (AGO: 0.40 ± 0.07, *P* = 0.0007; AGO: 0.41 ± 0.06, *P* = 0.022; AGO: 0.56 ± 0.08, *P* = 0.0003; AGO: 0.29 ± 0.04, *P* < 0.0014; AGO: 0.42 ± 0.12, *P* = 0.0253; AGO: 0.30 ± 0.05, *P* < 0.0001; AGO: 0.48 ± 0.11, *P* = 0.0297). The results provided evidence that agomelatine may exert a neuroprotective effect by mediating DHCR24, Akt/mTOR/p70s6k, and Hes1/Notch1 signaling events.

## Discussion

As the most prevalent neurogenerative disease causing dementia, the etiology of AD is not clear. Amyloid-β deposition and tau hyperphosphorylation are always considered the core pathological damage in AD (Scheltens et al., 2016). In recent years, more studies demonstrated that neuroinflammation, characterized by the activation of microglia and astrocyte, played an essential role in the onset and development of AD (Kaur et al., 2019). Besides, along with the procession of AD, microglial polarization was markedly transformed from anti-inflammatory M2 to pro-inflammatory M1 phenotype, resulting in the secretion of abundant pro-inflammatory cytokines such as IL-1β and TNF-α (Yao and Zu, 2020). Persistent and excessive neuroinflammation elicited detrimental effects bringing neuronal injury in the affected cerebral region (Kaur et al., 2019). In line with previous studies, we found that the level of pro-inflammatory cytokines (IL-1β and TNF-α) obviously increased, and the anti-inflammatory cytokines (IL-4) decreased in the hippocampus tissue of APP/PS1 mice. Moreover, the protein expression of astrocyte marker (GFAP) and M1 microglial marker (i-NOS) was upregulated, while the expression of M2 microglial biomarker (arginase-1) was downregulated. In contrast, as a growth factor, TGF-β1 exhibited anti-inflammatory and neuroprotective properties against Aβ deposition and neurodegeneration in AD (Caraci et al., 2018). The deficit of TGF-β1 signaling was critically involved in the pathophysiology of AD, and the role of TGF-β1 is quite complex in AD (Caraci et al., 2018). Previous studies proved that the content of cerebral TGF-β1 was elevated in the AD mouse model (Das and Golde, 2006), and overexpressing TGF-β1 could aggravate Aβ deposition and AD-like vascular damage in mouse (Wyss-Coray et al., 1997; Ongali et al., 2010). In this article, we showed an elevation of TGF-β1 expression in APP/PS1 mice. Overall, the results suggested that the obvious inflammatory response and aberrant TGF-β1 signaling may be essentially implicit in the etiology of AD.

As a new type of anti-depressant, agomelatine is a muti-receptor agonist including MT1 and MT2 melatonin receptors and a selective 5-HT 2C receptor antagonist, which is widely used in psychological symptoms treatment, such as depression, insomnia, and apathy in several neurodegenerative diseases (Green, 2011; Liu et al., 2016; Ilieva et al., 2019). However, less is known about the effect of agomelatine in AD therapy. A clinical case showed that in addition to insomnia, cognition, and depression symptoms, daily function and MMSE score were also improved by 1-month agomelatine treatment in a 91-years AD patient (Altınyazar and Kiylioglu, 2016). Our previous study also demonstrated that in Aβ_25–35_-treated BV-2 cells, tau protein hyperphosphorylation and oxidative injury could be suppressed by agomelatine intervention, which suggested agomelatine may provide a neuroprotective effect in AD (Yao et al., 2019). Kalina Ilieva et al. (2019) reported that in the streptozotocin (STZ)-induced AD rat model, agomelatine injection could decrease Aβ protein expression in the hippocampal region and, moreover, improve the anxiety-like behavior and spatial memory performance of rats. Although less evidence has focused on the effect of agomelatine in AD, there are also limitations. First, the effect of agomelatine on AD-like pathology, including tau protein hyperphosphorylation and neuroinflammation, is still not clear. Second, the protective molecular mechanisms of agomelatine in AD therapy are not fully understood.

To conclude, we first studied the effect of agomelatine on AD-like pathology in the AD transgenic mouse model. After agomelatine treatment, the Aβ and P-tau expression in the hippocampus of APP/PS1 mice were significantly declined; subsequently, the cognitive deficits of mice exhibited in the MWM were improved. Then, the anti-inflammatory activity of agomelatine was studied. The previous report revealed that agomelatine intervention could alleviate isoflurane-induced inflammation by decreasing the level of IL-6, IL-8, TNF-α, VCAM-1, and ICAM-1 in brain endothelial cells (Cheng et al., 2020). Hans O. Kalkman also indicated that agomelatine suppressed inflammation and M1-microglial polarization *via* modulating IGF1 expression (Kalkman and Feuerbach, 2016). In LPS-treated rats, agomelatine injection could ameliorate inflammatory response by inhibiting the production of pro-inflammatory cytokines (NF-kB and IL-10) (Savran et al., 2020). However, the effect of agomelatine on AD-related neuroinflammation is not well known. Our results suggested that in the AD mouse model, agomelatine injection could ameliorate the inflammatory response, probably by diminishing the level of pro-inflammatory cytokines, normalizing astrocyte activation, and promoting microglial polarization from M1 to M2 phenotype. In contrast, it was reported that rescuing TGF-β signaling could alleviate the pathological injury like Aβ deposition in AD, which was intended to be a potential therapeutic target of AD (Town et al., 2008; Caraci et al., 2018). This study showed that agomelatine could rescue TGF-β1 expression in APP/PS1 mice. The novel results served as proof that agomelatine had great potential to prevent the pathological damage and development of AD. These studies thus are expected to offer a new strategy to treat AD in the future.

Furthermore, the underlying molecular mechanism of agomelatine was discussed. 3β-Hydroxysterol Δ(24)-reductase (DHCR24), also named selective AD indicator-1, plays a vital role in the process of cholesterol synthesis and homeostasis (Drzewińska et al., 2009). In addition, DHCR24 importantly regulates a series of cellular functions such as membrane lipid-raft formation, cell apoptosis, neurosteroids activity, and oxidative stress (Peri et al., 2009; Zerenturk et al., 2013). Lots of evidence showed the close link between DHCR24 and AD. Hosseinzadeh et al. (2015) found the downregulation of DHCR24 accompanied by cognitive impairment in STZ-induced AD rat model. Besides, knocking out DHCR24 was reported to aggravate Aβ production and accumulation in the AD mouse model (Crameri et al., 2006), and overexpressing DHCR24 could decrease Aβ levels and inhibit Aβ toxicity *in vitro* (Sarajärvi et al., 2009). Bai et al. (2021) indicated that DHCR24 knock-down induced tau protein hyperphosphorylation in SH-SY5Y cells. Another study showed a low serum DHCR24 protein level in AD patients with diabetes mellitus (DM) (Önmez et al., 2020). Moreover, previous few studies showed the anti-inflammatory effect of DHCR24. Wang et al. (2019) demonstrated that DHCR24 was critically involved in the anti-inflammatory activity of apolipoprotein M (ApoM) *in vivo* and *in vitro*. We also found that DHCR24 over-expression could alleviate inflammation by promoting microglial polarization in Aβ_25–35_-treated BV-2 cells (Zu et al., 2020). This article provided a novel report that the hippocampal DHCR24 expression was significantly upregulated by agomelatine injection in APP/PS1 AD model. In our opinion, agomelatine may modulate DHCR24 activity through multiple mechanisms, including (1) potentially regulating the sterol regulatory element-binding protein (SREBP) expression (Joshi et al., 2021) and cholesterol metabolism (Hong et al., 2020), and previous studies supported a close link between SREBP activity, lipid homeostasis, and DHCR24 expression (Kallin et al., 2007; Ramos et al., 2012) and (2) affecting the production of a series of hormones such as sex steroids (Cipolla-Neto et al., 2021) and hypothalamic-pituitary-adrenal axis (Tchekalarova et al., 2016), which are considered to correlate with the expression of the DHCR24 (Zerenturk et al., 2013). The underlying mechanism is less known and needs to be further studied. In this study, we described a new protective mechanism of agomelatine in AD and revealed the potential link between antidepressant agomelatine and cholesterol metabolism.

The previous studies revealed the critical role of autophagy in a series of biological processes, including energy metabolism, cellular proliferation, and survival (Levine and Klionsky, 2004; Heras-Sandoval et al., 2020). As the primary regulator of autophagy, the activity of kinase mammalian target of rapamycin (mTOR) is modulated by numerous stimuli like neurotransmitters, hormones (Cuyàs et al., 2014), amino acids (Wolfson et al., 2016), glucose (Masui et al., 2015), and phosphatidic acid (PA) (Menon et al., 2017). It is reported that PI3k/Akt signaling activation could promote mTOR expression (Laplante and Sabatini, 2012), resulting in decreased lysosomal biogenesis and downstream autophagy-related genes expression, and finally inhibiting cellular autophagy (Yang and Klionsky, 2010). Accumulating evidence suggested that mTOR signaling inhibitors exerted a neuroprotective effect in AD (Yao and Zu, 2020). Inhibiting Akt/mTOR signaling by diverse compounds, such as IFN-γ injection (He et al., 2020), geniposide (Zhang et al., 2020), curcumin (Wang et al., 2014), could markedly induce Aβ clearance, alleviate cerebral injury, and improve the behavioral ability in APP/PS1 mice (Heras-Sandoval et al., 2020). Besides, targeting mTOR signaling by mTOR inhibitor could potently suppress the excessive neuroinflammation by promoting the microglial phenotype switch from M1 to M2 phenotype, lowering the secretion of pro-inflammatory cytokines (Luo et al., 2018; Yao and Zu, 2020), and reversing the astrocyte activation (Ou et al., 2018). In our study, Western blot analysis showed that agomelatine could inhibit Akt/mTOR signaling by downregulating the protein expression of P-Akt, P-mTOR, and P-p706sk in the hippocampus of APP/PS1 mice. Due to the close relationship between Akt/mTOR signaling pathway and AD, we novelly concluded that agomelatine might provide a protective function in AD by inhibiting Akt/mTOR signaling.

In contrast, Notch signaling was considered to be importantly implicated in neuronal development, angiogenesis, memory formation, and synaptic plasticity. Aberrant Notch signaling was closely related to the pathophysiology of several neurogenerative diseases, including AD (Ho et al., 2020), and the overactivation of Notch1 signaling induced by brain damage is harmful to neuronal survival (Brai et al., 2016). A previous study identified that Notch1 expression was obviously upregulated in the brains of AD patients (Berezovska et al., 1998). Furthermore, there is a close interaction between the amyloid precursor protein (APP) pathway and Notch signaling (Ho et al., 2020). Galeano et al. (2018) utilized lentiviral particles (LVP) to express the transcriptionally active fragment of Notch in the hippocampus of AD rat model. Subsequently, they found that the performance of MWM and the Aβ deposition in the hippocampal vessel were worsened (Galeano et al., 2018). Another study found that Osthole, a natural coumarin derivative, could restore cognitive functions and reduce Aβ deposition in APP/PS1 mice, probably by inhibiting Notch signaling (Li et al., 2017). Chen et al. (2019) reported that MicroRNA-98 reduced Aβ production and ameliorated oxidative stress injury through inactivating the Notch signaling pathway in AD mice. Moreover, abundant evidence showed that Notch signaling blockage exhibited anti-inflammatory properties and promoted microglial polarization from M1 to M2 phenotype *in vitro* and *in vivo* (Cheng et al., 2019; Yao and Zu, 2020). In this study, we showed that agomelatine could reverse the activation of Hes1/Notch1 signaling in APP/PS1 mice. The abovementioned data contribute to revealing the neuroprotective mechanism of agomelatine in the AD model and provide a novel promising therapeutic target for AD drug development.

## Conclusion

Our study demonstrated that antidepressant agomelatine could inhibit Aβ deposition, tau protein phosphorylation, and neuroinflammatory response in the hippocampus of APP/PS1 mice. Agomelatine may exert an anti-inflammatory effect *via* promoting microglial polarization from M1 to M2 phenotype and suppressing astrocyte activation. Furthermore, the neuroprotective effect of agomelatine may be through activating DHCR24 signaling and inhibiting Akt/mTOR signaling and Hes1/Notch1 signaling. The abovementioned data identified the neuroprotective function of agomelatine in the AD mouse model and contributed to providing a new target for AD therapy.

## Data Availability Statement

The original contributions presented in the study are included in the article/supplementary material, further inquiries can be directed to the corresponding author.

## Ethics Statement

The animal study was reviewed and approved by Shanghai Public Health Clinical Center Laboratory Animal Welfare and Ethics Committee.

## Author Contributions

X-BY, H-BZ, Y-FZ, and KY were responsible for the experimental operation, data statistics, and manuscript drafting of the study. KY conceived the initial idea, designed the study, and participated in the manuscript revision. All authors read and approved the final manuscript.

## Conflict of Interest

The authors declare that the research was conducted in the absence of any commercial or financial relationships that could be construed as a potential conflict of interest.

## Publisher’s Note

All claims expressed in this article are solely those of the authors and do not necessarily represent those of their affiliated organizations, or those of the publisher, the editors and the reviewers. Any product that may be evaluated in this article, or claim that may be made by its manufacturer, is not guaranteed or endorsed by the publisher.
